# Retrospective Assessment of Risk Factors for Head and Neck Cancer Among World Trade Center General Responders

**DOI:** 10.3389/fpubh.2020.488057

**Published:** 2020-11-30

**Authors:** Michelle T. Bover Manderski, Kathleen Black, Iris G. Udasin, Taylor M. Black, Michael B. Steinberg, Anna R. Giuliano, Benjamin J. Luft, Denise Harrison, Michael A. Crane, Jacqueline Moline, Marian R. Passannante, Pamela Ohman Strickland, Christopher R. Dasaro, Roberto G. Lucchini, Andrew C. Todd, Judith M. Graber

**Affiliations:** ^1^Department of Biostatistics and Epidemiology, Rutgers School of Public Health, Piscataway, NJ, United States; ^2^Environmental and Occupational Health Sciences Institute, Rutgers, The State University of New Jersey, Piscataway, NJ, United States; ^3^Division of General Internal Medicine, Robert Wood Johnson Medical School, Rutgers, The State University of New Jersey, New Brunswick, NJ, United States; ^4^Center for Immunization and Infection Research in Cancer, Moffitt Cancer Center, Tampa, FL, United States; ^5^Department of Medicine, Stony Brook University Medical Center, Stony Brook, NY, United States; ^6^Department of Environmental Medicine, Bellevue Hospital Center/New York University School of Medicine, New York, NY, United States; ^7^Department of Environmental Medicine and Public Health, Icahn School of Medicine at Mount Sinai, New York, NY, United States; ^8^Department of Occupational Medicine, Epidemiology and Prevention, Hofstra Northwell School of Medicine at Hofstra University, Hempstead, NY, United States

**Keywords:** behavioral risk factors, cancer, questionnaire, reliability, World Trade Center (WTC)

## Abstract

**Objective:** To assess the reliability of a questionnaire designed to reconstruct risk factors for head and neck cancer relative to the 9/11 World Trade Center (WTC) response and over the lifetime.

**Methods:** As part of a nested case-control study, 200 WTC Health Program (WTCHP) General Responder Cohort (GRC) members completed a newly-developed study questionnaire *via* telephone (with a trained interviewer) or online (self-administered). We assessed agreement between measures of tobacco and alcohol use in our questionnaire results and data collected previously during WTCHP-GRC monitoring visits using Cohens Kappa (κ) and intraclass correlation coefficient (ICC) for categorical and continuous measures, respectively. We compared agreement by disease status, survey mode, and year of WTCHP enrollment.

**Results:** We observed high agreement between measures of lifetime, pre-WTC, and post-WTC smoking prevalence (all κ > 0.85) and smoking duration (all ICC > 0.84). There was moderate agreement between measures of smoking frequency (ICC: 0.61–0.73). Agreement between measures of smoking frequency, but not duration, differed by disease status, and agreement between smoking measures was higher for participants who completed our survey by phone than by web. Among cases, there were no differences based on enrollment in the WTCHP before or after diagnosis.

**Conclusion:** Agreement between measures was generally high, although potential reporting bias and a mode effect that should be considered when interpreting analyses of self-reported data in this population; however differential misclassification appears to be minimal. Our questionnaire may be useful for future studies examining similar behavioral risk factors among disaster-exposed populations.

## Introduction

People involved in the World Trade Center (WTC) rescue, recovery, and cleanup efforts following the collapse of the WTC towers on September 11, 2001 (9/11) faced potential exposure to multiple known and suspected human carcinogens (1–3). As such, WTC-related exposures may place responders at increased risk for adverse health outcomes, including cancer. Indeed, studies have found excess incidence of “all cancers” and certain cancer sites among WTC-exposed persons, including cancers of the prostate and thyroid (4, 5). As well, a 40% excess incidence [standardized incidence ratio (SIR): 1.40, 95% confidence interval (CI): 1.01, 1.89] of head and neck cancers diagnosed between 2009 and 2012 has been reported among members of the WTC Health Program (WTCHP) General Responder Cohort (6). However, other population-level behavioral risk factors, including tobacco use, alcohol consumption, and increased sexual activity [an established indicator of human papillomavirus (HPV) infection risk (7–9)] (10–14), may play a role in the etiology of head and neck cancer in this population. Given that risk behaviors may change over time or after traumatic events (such as participation in the WTC response), reconstruction of these risk factors before, during, and after the WTC exposure period, is critical to understanding any associations between WTC exposures and head and neck cancer.

The WTC Health Program provides medical monitoring to eligible WTC responders and treatment for certified conditions (15). At enrollment, General Responder Cohort members receive an initial physical examination and are eligible for annual health monitoring visits thereafter. Data collected at monitoring visits include some limited information on tobacco and alcohol use.

The WTC Health Program General Responder Data Center (GRDC) in the Icahn School of Medicine at Mount Sinai maintains monitoring data collected from General Responder Cohort members. This includes objective clinical data and self-reported data on WTC exposure, physical and mental health symptoms, and behaviors that, with consent, can be available for research purposes. However, as a medical monitoring program, the WTC Health Program collects general information about common health-related behaviors (smoking and alcohol use) but does not contain the detailed information necessary to construct accurate lifetime behavioral risk factors. For example, most questions about alcohol use concerned current consumption, making it difficult to reconstruct this risk behavior over ones lifetime. Moreover, several potential risk factors for head and neck cancer, including smokeless tobacco, marijuana use, and sexual behavior, were not assessed, further necessitating the need to develop a retrospective risk factor assessment tool.

As part of a larger nested case-control study of head and neck cancer among WTC Health Program members (WTC Cancer Risk Epidemiology Study, WTC-CARES) (16), we developed a risk factor assessment questionnaire designed to reconstruct lifetime exposure to tobacco, alcohol, and marijuana use, as well as tooth loss and sexual history (as a surrogate measure of HPV risk). Although we developed questions based on previously validated instruments, to the extent possible it is important to assess validity and reliability of study questionnaires in each population. As such, the overall objective of this methods study was to describe development and reliability testing of a new retrospective questionnaire designed to measure detailed behavioral risk factors among responders to the WTC disaster.

## Materials and Methods

### Study Population

Data were collected from participants of WTC-CARES, a nested case-control study of WTC Health Program General Responder Cohort members (16). Eligible cases were members diagnosed with head and neck cancer between 2002 and 2016 [International Classification of Disease (ICD)-9 codes 140-149.9, 160-161.0; ICD-10 codes C00.0-C14.9, C32-C32.9]. We identified 102 cases, of whom 94 were eligible (i.e., living and consented to be contacted for WTC-related research) and 64 (68.1%) consented to participate. Controls (*n* = 136) were cohort members without cancer, identified *via* risk-set sampling and then individually matched 2-to-1 on age, sex, and race/ethnicity.

### The WTC Cancer Risk Epidemiology Study (WTC-CARES) Questionnaire

Development of the WTC-CARES questionnaire was informed by standardized survey questions (17–19), as well as by a literature review of other studies that assessed head and neck cancer risk factors *via* questionnaire (20, 21). Although informative in terms of question content and structure, no previous studies had attempted to reconstruct lifetime risk behaviors at periods relative to a specific event such as the WTC response period. Adaptation, review, and testing of the resulting study instrument were therefore critical.

We assessed face and content validity, as well as cultural appropriateness, through expert review and cognitive interviews with members of the target population. Reviewers were colleagues and/or collaborators of the study investigators and included: two WTC Health Program clinicians; a medical internist and tobacco treatment expert; a cancer epidemiologist and HPV expert; two oncologists; two cancer epidemiologists from the New Jersey State Cancer Registry (NJSCR); an occupational epidemiologist; and a survey methodologist. We then tested the questionnaire *via* cognitive interviewing, a technique used to study the process through which a respondent interprets a question and formulates a response. Specifically, a cognitive interview assesses question comprehension (how the respondent interprets the question), recall (how the respondent searches memory for relevant information), judgement (how the respondent evaluates and estimates the response), and response (does the respondent provide information in the format requested) (22). Interviews occur in “rounds” such that after completing several interviews, the questionnaire is revised based on findings and retested in subsequent rounds with different participants. This process continues until no additional problems are identified (23). Though there is no set rule, typical cognitive interviewing protocols suggest 3–4 iterative rounds of 5–10 interviews each. However, given limited resources, we employed an abbreviated approach similar to that described by Spark and Willis (24). We conducted 4 rounds of 3 interviews each for a total of 12 interviews. Interview participants included three cohort members who had cancer (other than head and neck cancer), six cohort members without cancer, and three non-WTC-exposed cancer patients. We recruited cohort participants from the WTC Clinical Center of Excellence (CCE) at Rutgers in Piscataway, New Jersey and non-WTC-exposed participants from the Rutgers Tobacco Dependence Program in New Brunswick, New Jersey.

The final WTC-CARES questionnaire assessed tobacco use (cigarette, cigar, smokeless tobacco, hookah, and pipe), alcohol use, marijuana use, and environmental/occupational exposures (e.g., asbestos and dusts) during each of three time periods: (1) before September 11th, 2001, (2) during the time the participant worked or volunteered on the WTC response efforts, and (3) subsequently until the time of cancer diagnosis (for controls, this was the date of diagnosis for the matched case). Additionally, measures of oral health (i.e., tooth loss before and after 9/11 and tonsillectomy), sexual history (i.e., age of sexual debut, number of sexual partners during each of the three time periods, history of a sexually transmitted infection, and receipt of HPV vaccine), and mental health treatment (i.e., years of treatment episodes) were included.

### The WTC Health Program Questionnaires

WTC Health Program General Responder Cohort members receive an annual health monitoring exam, which includes a clinical exam as well as self-reported assessment of physical, mental, and behavioral health (15). At the initial visit, questionnaires include items about: occupational and environmental exposures during participation in the WTC response and as associated with other occupations or hobbies; lifetime cigarette smoking (i.e., whether ever smoked, age first smoked, whether currently smoking, age last smoked, and cigarettes per day); ever and current cigar smoking; ever and current pipe smoking; and current alcohol consumption. The periodic questionnaires, completed at subsequent visits, include assessments of: occupational and environmental exposures (since previous exam); lifetime cigarette smoking (for members who were never or former smokers at previous exam: ever smoker, current smoker, age last smoked, and cigarettes per day); current cigarette smoking; ever and current cigar smoking; ever and current pipe smoking; and current alcohol consumption.

### Data Collection

The Health Sciences Institutional Review Board at Rutgers University reviewed and approved the study protocol, including recruitment, consent, and data collection procedures.

Potential participants were mailed a letter with information about the study and how to schedule a telephone interview or complete the survey online. Trained interviewers administered our survey by telephone; alternatively, participants could complete the survey online. Though not the preferred mode of data collection, we offered a web-based option, because sequelae of head and neck cancer or treatment can include speech impairment. We took substantial care to minimize differences between survey modes. For example, the interviewer- and self-administered surveys were identical with respect to text and supplemental information, and the interviewers were trained to avoid script deviations. Data collection occurred from July 2017 through April 2018.

We obtained deidentified data from the WTC Health Program General Responder Data Center for all WTC-CARES participants *via* a data use agreement. For participants who enrolled in the cohort prior to cancer diagnosis (for controls, this was the date of diagnosis for the matched case), we included data from all monitoring visits up to and including the year of diagnosis for reconstruction of risk behaviors. For participants who enrolled after the cancer diagnosis, we considered only data from the first monitoring visit. WTC Health Program data included for this analysis were collected between 2002 and 2016 (median 2007).

### Measures

#### Tobacco Use

Both our WTC-CARES and the WTC Health Program questionnaires assessed lifetime ever cigarette smoking by asking participants if they had smoked at least 100 cigarettes. For WTC-CARES, we separately assessed smoking prior to, during, and after the WTC responses, thus determining smoking status (“ever,” “current,” “former,” and “never”), as well as “duration” and “frequency” of smoking during each study period based on responses to the period-specific questions (hereafter, these terms, as well as others like them, will be used without quotes). For each study period, we also asked if there were a period of more than 1 year during which the participant did not smoke at all, accounting for this information when calculating duration of smoking. For the WTC Health Program data, we inferred ever and duration of smoking during each study period based on the age of smoking initiation provided at visit one and the smoking status (and age of smoking cessation, if applicable) at the monitoring visit closest to (but not exceeding) the year of cancer diagnosis. Because information about changes in smoking frequency over the lifetime was not collected, we assumed a constant smoking frequency (i.e., cigarettes per day). For example, if a participant was 40 in 2001 and reported smoking 20 cigarettes per day at the monitoring visit in 2015, we assumed they had been smoking 20 cigarettes per day for 14 years during the post-WTC study period. We also descriptively assessed changes in smoking status over time.

#### Alcohol Consumption

As with tobacco use, our WTC-CARES questionnaire separately assessed alcohol consumption during each of the three study periods and determined ever, duration, and frequency of drinking, as well as frequency of binge drinking [defined as five or more (for men, four or more for women) drinks in a single day]. For the WTC Health Program, only current alcohol consumption was consistently assessed at baseline and follow-up visits, thus ever drinking during the post-WTC study period was inferred when a participant indicated any alcohol consumption during an applicable monitoring visit (i.e., up to and including year of cancer diagnosis). Although the WTC Health Program questionnaire did assess frequency of drinking (drinks per week), substantial missing data appreciably limited their utility for analysis; thus, only ever/never current consumption could be inferred.

### Statistical Analysis

In the absence of a gold standard, we assessed agreement between our WTC-CARES and the WTC Health Program measures for each construct assessed by both data sources (i.e., ever, duration, and frequency of cigarette smoking before, during, and after WTC exposure; ever alcohol consumption after WTC exposure). For categorical measures we estimated agreement using Cohen's Kappa (κ) statistic (25), considering estimates <0, 0–0.2, 0.21–0.4, 0.41–0.6, 0.61–0.8, and >0.8 indicative of “poor,” “slight,” “fair,” “moderate,” “substantial,” and “near perfect” agreement, respectively (26). For continuous measures, we estimated agreement using intraclass correlation coefficient (ICC), employing absolute agreement two-way mixed models and considering estimates <0.5, 0.5 to >0.75, 0.75 to <0.9, and >0.9 indicative of “poor,” “moderate,” “good,” and “excellent” reliability (27, 28). For risk factor measures, we further compared agreement estimates by case/control status, and, among cases, year of WTC Health Program enrollment (before vs. after year of diagnosis) with non-overlapping 95% confidence intervals considered indicative of statistical significance. Additional sensitivity analyses included comparison of agreement of behavioral risk factor measures by WTC-CARES survey mode (telephone vs. web), by enrollment before 2007 (median year of enrollment) vs. 2007 or later, and occupation. To assess potential selection bias, we also compared risk behaviors, as measured by the WTC Health Program, for cases who enrolled in WTC-CARES and cases who did not enroll (*n* = 38, including 8 deceased cases), using two-sided chi-square-tests and *t*-tests for categorical and continuous measures, respectively.

We performed all analysis using SAS 9.4 (SAS Institute, Cary, North Carolina, USA) and SPSS 24 (IBM Corp., Armonk, New York, USA) software packages.

## Results

Two hundred WTC-CARES participants, including 64 cases and 136 controls, contributed to this analysis. Most participants were male (88.5%) and on average 41.7 years old [standard deviation (SD): 6.8] on 9/11 and 48.2 years old (SD: 8.3) at enrollment in the WTC Health Program (Table 1). Cases were slightly older than controls at enrollment in the WTC Health Program, but gender distributions were similar across study groups. The majority of participants were non-Hispanic white (69.2%) and had protective services occupations (51.0%).

**Table 1:** Participant demographics, WTC cancer risk epidemiology study, *N* = 200.

	**Overall**	**Cases**	**Controls**
	***n* = 200**	***n* = 64**	***n* = 136**
Age on 9/11, mean ± SD	41.7 ± 6.8	41.9 ± 6.8	41.6 ± 6.8
Age at enrollment in WTCHP, mean ± SD	48.2 ± 8.3	51.1 ± 9.1	46.8 ± 7.5
Male sex, *n* (%)	177 (88.5)	57 (89.1)	120 (88.2)
Race/ethnicity, *n* (%)			
Non-Hispanic white	137 (69.2)	52 (82.5)	85 (63.0)
Non-Hispanic black	24 (12.1)	^*^	^*^
Hispanic	31 (15.7)	8 (12.7)	23 (17.0)
Non-Hispanic other	5 (2.5)	0	5 (3.7)
Occupation, *n* (%)			
Protective services	102 (51.0)	39 (60.9)	63 (46.3)
Construction	34 (17.0)	8 (12.5)	26 (19.1)
Mechanic/machinist	8 (4.0)	^*^	^*^
Communications	16 (8.0)	5 (7.8)	11 (8.1)
Other	40 (20.0)	11 (17.2)	29 (21.3)

*WTC, World Trade Center; SD, standard deviation*.

* *Cell counts <5 are suppressed according to terms of the data use agreement between Rutgers, The State University of New Jersey and the WTC Health Program General Responder Data Center*.

We observed near perfect agreement between ever smoking measures overall and during each study period (all κ > 0.85) and for overall, pre-WTC, and post-WTC years of smoking; however, we observed lower agreement for duration of smoking during the WTC response period (κ = 0.5; Table 2). With respect to smoking frequency (cigarettes per day) during each study period, we observed moderate agreement, with higher prevalence estimates from the WTC Health Program measures than our WTC-CARES measures. Descriptive analysis of WTC Health Program smoking status stratified by WTC-CARES smoking status found that 7 of 111 (6.3%) participants who reported “never smoking” in our WTC-CARES study had reported ever or former smoking during a WTC Health Program monitoring visit (data not shown). Among those who reported current smoking in our WTC-CARES study (*n* = 15), all reported the same behavior at a WTC Health Program monitoring visit.

**Table 2:** Agreement of risk factor measures as assessed by WTC-CARES and WTCHP, *N* = 200.

	**WTC-CARES**	**WTCHP**	**Agreement^a^**
	***n* (%)**	***n* (%)**	**Est. (95% CI)**
**Risk behaviors**			
**Ever cigarette smoking**, ***n*** **(%)**			
Overall	89 (44.5)	92 (46.0)	0.89 (0.83, 0.95)
Prior to WTC exposure	88 (44.0)	90 (45.0)	0.90 (0.84, 0.96)
During WTC exposure	34 (17.0)	42 (21.7)	0.85 (0.76, 0.95)
After WTC exposure	44 (22.0)	44 (22.0)	0.85 (0.77, 0.94)
**Years of cigarette smoking, mean** **±** **SD**			
Overall	8.4 ± 12.2	10.1 ± 14.5	0.88 (0.84, 0.91)
Prior to WTC exposure	7.1 ± 9.9	8.7 ± 11.7	0.91 (0.87, 0.94)
During WTC exposure	0.6 ± 0.2	0.1 ± 0.2	0.50 (0.37, 0.61)
After WTC exposure	1.4 ± 3.3	1.7 ± 3.9	0.84 (0.79, 0.87)
**Average cigarettes per day, mean** **±** **SD**			
Prior to WTC exposure	4.7 ± 8.7	7.0 ± 10.6	0.61 (0.50, 0.69)
During WTC exposure	2.3 ± 6.7	3.3 ± 7.9	0.57 (0.46, 0.66)
After WTC exposure	2.0 ± 6.4	3.4 ± 8.0	0.73 (0.65, 0.79)
**Ever alcohol drinking**, ***n*** **(%)**			
Overall	169 (84.5)	–	–
Prior to WTC exposure	167 (83.5)	–	–
During WTC exposure	152 (76.0)	–	–
After WTC exposure	154 (77.0)	143 (73.7)	0.51 (0.37, 0.65)

*WTC-CARES, World Trade Center Cancer Risk Epidemiology Study; WTCHP, World Trade Center Health Program; SD, standard deviation; CI, confidence interval*.

a *Agreement assessed by kappa (κ) statistic for categorical measures or intraclass correlation coefficient (ICC) for continuous measures*.

In contrast to that observed for smoking, WTC-CARES estimates for post-WTC alcohol drinking prevalence were higher (77.0 vs. 73.7%) and agreed moderately with WTC Health Program estimates (κ = 0.51). By occupational group, agreement between post-WTC alcohol measures was somewhat lower for those in the protective services as opposed to other occupations, although this difference was not statistically significant [κ = 0.42 (95%CI: 0.20, 0.64) vs. κ = 0.58 (95% CI: 0.40, 0.76), data not shown].

In general, we saw no differences in agreement for risk factors by disease status, with the exception of cigarette smoking frequency (Table 3). Agreement was significantly higher for cases when considering the pre-WTC study period [ICC = 0.80 (95% CI 0.67, 0.88) for cases vs. ICC = 0.55, (95% CI 0.41, 0.66) for controls] but significantly higher for controls when considering the post-WTC period [ICC = 0.52 (95% CI 0.36, 0.71) for cases vs. ICC = 0.82, (95% CI 0.75, 0.87) for controls].

**Table 3:** Agreement of risk factor measures as assessed by WTC-CARES and WTCHP, by case/control status, *N* = 200.

**Construct**	**Agreement (95% CI)^a^**	
	**Cases, *n* = 64**	**Controls, *n* = 136**
**Ever cigarette smoking**		
Overall	0.94 (0.85, 1.00)	0.86 (0.78, 0.95)
Prior to WTC exposure	0.94 (0.85, 1.00)	0.88 (0.80, 0.96)
During WTC exposure	0.88 (0.74, 1.00)	0.83 (0.70, 0.96)
After WTC exposure	0.85 (0.71, 0.99)	0.85 (0.74, 0.97)
**Years of cigarette smoking**		
Overall	0.89 (0.83, 0.93)	0.87 (0.82, 0.91)
Prior to WTC exposure	0.91 (0.84, 0.95)	0.91 (0.87, 0.94)
During WTC exposure	0.47 (0.23, 0.65)	0.52 (0.38, 0.64)
After WTC exposure	0.87 (0.79, 0.92)	0.81 (0.75, 0.86)
**Average cigarette per day**		
Prior to WTC exposure	0.80 (0.67, 0.88)	0.55 (0.41, 0.66)
During WTC exposure	0.60 (0.41, 0.74)	0.55 (0.42, 0.66)
After WTC exposure	0.56 (0.36, 0.71)	0.82 (0.75, 0.87)
**Ever alcohol drinking**		
After WTC exposure	0.52 (0.31, 0.74)	0.49 (0.30, 0.68)

*WTC-CARES, World Trade Center Cancer Risk Epidemiology Study; WTCHP, World Trade Center Health Program; CI, confidence interval*.

a *Agreement assessed by kappa (κ) statistic for categorical measures or intraclass correlation coefficient (ICC) for continuous measures*.

Agreement between measures of ever cigarette smoking overall and during each study period was substantial to near perfect for both survey modes (Table 4). For measures of smoking duration, agreement was generally higher for the telephone survey than the web survey, though none of these differences was statistically significant. Agreement was also higher for the telephone group when comparing measures of average cigarette consumption prior to and during WTC exposure, and these differences were statistically significant. There were no differences in agreement by survey mode for post-WTC alcohol consumption.

**Table 4:** Agreement between WTC-CARES and WTCHP measures of behavioral risk factors, by survey mode (*N* = 200).

**Construct**	**Agreement (95% CI)^a^**	
	**Web, *n* = 63**	**Phone, *n* = 137**
**Risk behaviors**		
**Ever cigarette smoking**		
Overall	0.94 (0.85, 1.00)	0.87 (0.78, 0.95)
Prior to WTC exposure	0.94 (0.85, 1.00)	0.88 (0.80, 0.96)
During WTC exposure	0.78 (0.58, 0.98)	0.88 (0.78, 0.98)
After WTC exposure	0.82 (0.64, 0.99)	0.87 (0.77, 0.97)
**Years of cigarette smoking**		
Overall	0.86 (0.77, 0.91)	0.89 (0.84, 0.92)
Prior to WTC exposure	0.87 (0.77, 0.93)	0.93 (0.89, 0.95)
During WTC exposure	0.33 (0.10, 0.53)	0.56 (0.42, 0.67)
After WTC exposure	0.79 (0.68, 0.87)	0.86 (0.80, 0.90)
**Average cigarette per day**		
Prior to WTC exposure	0.41 (0.19, 0.60)	0.71 (0.60, 0.79)
During WTC exposure	0.26 (0.02, 0.47)	0.67 (0.56, 0.75)
After WTC exposure	0.83 (0.73, 0.89)	0.65 (0.54, 0.74)
**Ever alcohol drinking**		
After WTC exposure	0.52 (0.23, 0.81)	0.50 (0.34, 0.66)

*WTC-CARES, World Trade Center Cancer Risk Epidemiology Study; WTCHP, World Trade Center Health Program; CI, confidence interval*.

a *Agreement assessed by kappa (κ) statistic for categorical measures or intraclass correlation coefficient for continuous measures*.

Comparing cases enrolled before vs. after cancer diagnosis, we saw no significant differences in agreement of behavioral risk factor measures (Supplementary Table 1). Agreement between measures of post-WTC smoking prevalence and during-WTC smoking frequency was significantly higher among those who enrolled in the WTC Health Program in 2007 or after, as opposed to before 2007 (Supplementary Table 2). There were no other differences in agreement when comparing by enrollment year. Among cases, WTC Health Program-assessed smoking and drinking did not differ by WTC-CARES study enrollment status (Supplementary Table 3).

## Discussion

As part of a case-control study of head and neck cancer within the WTC Health Program General Responder Cohort, we developed a questionnaire to retrospectively assess risk factors before, during, and after WTC exposure, and, using these measures, calculated lifetime risk factors. We compared tobacco and alcohol data from our WTC-CARES questionnaire to that collected previously by the WTC Health Program and found substantial to near perfect agreement between measures of ever smoking during all study periods. We also observed good to excellent agreement between measures of lifetime, pre-WTC, and post-WTC smoking duration and between measures of post-WTC smoking frequency. Agreement was fair to moderate for measures of smoking duration during the WTC response, smoking frequency during and after the WTC response, and ever alcohol consumption after the WTC response. These findings suggest that the other risk behavior measures included in our questionnaire but not by the WTC Health Program questionnaires (e.g., measures of sexual behavior and marijuana use) would be similarly reliable, and our questionnaire may be useful for other studies of disaster-exposed populations.

We expected high agreement for measures of ever smoking, given the nearly identical wording of the WTC-CARES and WTC Health Program ever-smoking questions; however, we did not anticipate that the Health Program measure would yield slightly higher smoking prevalence estimates. Potential explanations for this finding include increased stigma in recent years associated with cigarette smoking that may have left the WTC-CARES questions subject to under-reporting. Indeed, about 6% of participants who reported never smoking in our WTC-CARES study had reported ever smoking in the WTC Health Program, whereas all who reported current smoking in WTC-CARES also reported smoking in the WTC Health Program. Moreover, we observed higher agreement of lifetime smoking measures among those enrolled in 2007 or later than among those enrolled prior to 2007. Although these differences were not statistically significant, they suggest that social desirability bias may explain why our WTC-CARES questionnaire yielded slightly lower smoking estimates.

We expected some disagreement between measures of smoking duration. The WTC-CARES questionnaire separately assessed years of smoking during each study period and accounted for any period when a participant was not smoking, whereas smoking duration in the WTC Health Program data was inferred as continuous between ages of first and last use. This may explain the higher average durations estimated by the WTC Health Program data. We similarly expected less agreement between measures of smoking frequency during each study period, because only the WTC-CARES questionnaire asked about consumption during each period, while the WTC Health Program questionnaires asked only about current (for current smokers) or lifetime average (for former smokers).

We can offer two potential explanations for our observation of only fair to moderate agreement between measures of post-WTC alcohol drinking. First, the WTC Health Program consistently assessed only current (at the time of the monitoring visit) drinking; however a participant's reported drinking status at the time of a monitoring visit may not reflect the entire post-WTC (until case diagnosis) period. Second, participants, many of whom were law enforcement officers, may have underreported alcohol consumption during a monitoring visit. A sensitivity analysis finding somewhat lower agreement between our WTC-CARES and the WTC Health Program alcohol measures among those in the protective services supports this notion.

Given the potential for recall bias in case-control studies, establishing agreement between current and previously-administered self-reported risk behaviors may alleviate concerns about misclassification (29, 30). In our study, recall bias may exist if cases and controls experience differential recall of exposures or risk factor behaviors. In this instance, cases might report their past behaviors differently when surveyed before as opposed to after diagnosis. When comparing WTC-CARES responses to pre-diagnosis WTC Health Program data among cases, we found substantial-to-near-perfect agreement between ever smoking and drinking measures, which may alleviate concerns about recall bias in our case-control study.

While there were no differences in agreement by survey mode for ever and years of smoking, we observed higher agreement for two cigarette smoking frequency measures among those who completed WTC-CARES by telephone, suggesting that certain analyses of WTC-CARES data may be subject to a mode effect. As such, future analysis of these data should include an assessment of survey mode and adjust for it if necessary.

There are several limitations to note. Reconstruction of WTC Health Program risk behavior data relative to the WTC exposure period was challenging, because some key information was not collected (e.g., changes in tobacco use frequency over the lifetime). Decisions made when creating equivalent constructs in each dataset may have contributed to differences between estimates produced by the WTC Health Program questionnaire and our WTC-CARES questionnaire. Whereas our questionnaire specifically asked about behavior frequency and duration during each study period (before, during, and after the WTC response), the WTC Health Program asked only about lifetime or current behaviors. As such, we inferred behavior for each study period based on peripheral information. However, finding that cumulative risk behaviors cannot be easily or adequately reconstructed relative to the WTC response period demonstrates the necessity of developing our own questionnaire for WTC-CARES.

Additionally, our results should be considered taking into account the small sample size, which resulted in wide confidence intervals when comparing agreement estimates by subgroup. This suggests that even null findings may be the result of inadequate power, rather than lack of association. Potential selection bias is of additional concern, if the distribution of head and neck cancer risk factors differs among people who enrolled in WTC-CARES and those whom we selected but did not enroll (e.g., refused). However, we found no differences in smoking or alcohol consumption by WTC-CARES enrollment status, suggesting minimal selection bias in the present analysis. Finally, we were not able to assess agreement for measures of other risk factors assessed by the WTC-CARES questionnaire (e.g., smokeless tobacco use, sexual behavior, and heavy alcohol consumption), because they were either not assessed by the WTC Health Program or had too many missing responses. However, it is not unreasonable to believe that the reliability we did observe likely extends to other measures we could not test.

Despite these limitations, our study has important findings. By demonstrating high reliability with smoking measured prior to diagnosis, we alleviate some concerns for recall bias in studies using this questionnaire. We also found some evidence of reporting bias in this population, as well as a potential mode effect, which we should be considered when interpreting results of studies among WTC-exposed populations. Finally, our results demonstrate that while the WTC Health Program monitoring data includes comprehensive WTC exposure information, assessment of lifetime risk behaviors among WTC-exposed persons requires additional measures. Our questionnaire may be useful for other studies of cancer outcomes in WTC-exposed populations. Moreover, findings from this methods study can inform questionnaire development and results interpretation for other research among disaster-exposed populations, which can assist with identification of higher-risk individuals and, by extension, improve detection of disease and treatment outcomes among people involved in disaster responses.

## Data Availability Statement

The datasets generated for this study will not be made publicly available. Primary data collected for this study (WTC-CARES data) may be available by reasonable request to the senior author (Graber); however, requests for the secondary data analyzed for this study (WTCHP monitoring data) may also require approval from the WTC General Responder Data Center.

## Ethics Statement

The studies involving human participants were reviewed and approved by Health Sciences Institutional Review Board at Rutgers, The State University of New Jersey, New Brunswick, New Jersey, USA. The Ethics Committee waived the requirement of written informed consent for participation.

## Author Contributions

MB, KB, MS, AG, and JG contributed to conception and design of the study. MB led questionnaire development in collaboration with KB, TB, IU, MS, AG, MP, and JG. TB organized the database. MB performed statistical analysis and wrote the first draft of the manuscript with guidance from JG. IU, BL, DH, MC, and JM led GRC data collection and contributed to manuscript writing. CD, RL, and AT maintained and provided GRC data and contributed to manuscript writing. All authors contributed to manuscript revision, read and approved the submitted version.

## Conflict of Interest

The authors declare that the research was conducted in the absence of any commercial or financial relationships that could be construed as a potential conflict of interest. The reviewer LC declared a shared affiliation, with no collaboration, with several of the authors, MB, KB, IU, TB, MS, MP, PO, and JG to the handling editor at the time of review.
