# Supplementary material for: Retrospective Assessment of Risk Factors for Head and Neck Cancer Among World Trade Center General Responders
Source: Front Public Health. 2020 Nov 30;8:488057. doi: 10.3389/fpubh.2020.488057 (PMC7734028; doi:10.3389/fpubh.2020.488057)
Supplement: Supplementary file 1 [file Table_1.PDF]

Supplementary Table 1: Agreement between WTC-CARES and WTCHP measures of select risk factors, among cases, by enrollment before vs. after diagnosis (N=64)

	Agreement (95% CI) [a]			
Construct	Enrolled Before Dx, n=36		Enrolled After Dx, n=27	
Risk Behaviors				
Ever Cigarette Smoking				
Overall	0.94	(0.83, 1.00)	0.93	(0.78, 1.00)
Prior to WTC exposure	0.94	(0.83, 1.00)	0.93	(0.78, 1.00)
During WTC exposure	0.93	(0.80, 1.00)	0.79	(0.51, 1.00)
After WTC exposure	0.87	(0.70, 1.00)	0.82	(0.59, 1.00)
Years of Cigarette Smoking				
Overall	0.85	(0.80, 0.89)	0.91	(0.84, 0.95)
Prior to WTC exposure	0.92	(0.88, 0.94)	0.88	(0.79, 0.93)
During WTC exposure	0.57	(0.45, 0.67)	0.38	(0.12, 0.59)
After WTC exposure	0.79	(0.72, 0.84)	0.88	(0.80, 0.93)
Average Cigarette per Day				
Prior to WTC exposure	0.54	(0.41, 0.65)	0.78	(0.62, 0.87)
During WTC exposure	0.54	(0.42, 0.65)	0.62	(0.42, 0.76)
After WTC exposure	0.77	(0.69, 0.83)	0.63	(0.43, 0.77)
Ever Alcohol Drinking				
After WTC exposure	0.69	(0.45, 0.94)	0.21	(-0.17, 0.60)

Note: WTC-CARES, World Trade Center Cancer Risk Epidemiology Study; WTCHP, World Trade Center Health Program; CI, confidence interval;
[a] Agreement assessed by kappa (κ) statistic for categorical measures or intraclass correlation coefficient for continuous measures

Supplementary Table 2: Agreement between WTC-CARES and WTCHP measures of behavioral risk factors, by enrollment before vs. after 1/1/07 (N=200)

	Agreement (95% CI) [a]			
Construct	Enrolled Before 2007 (n=95)		Enrolled in 2007 or later (n=105)	
Risk Behaviors				
Ever Cigarette Smoking				
Overall	0.85	(0.75, 0.96)	0.92	(0.85, 1.00)
Prior to WTC exposure	0.89	(0.80, 0.98)	0.90	(0.82, 0.99)
During WTC exposure	0.86	(0.72, 0.99)	0.85	(0.72, 0.98)
After WTC exposure	0.78	(0.63, 0.94)	0.92	(0.83, 1.00)
Years of Cigarette Smoking				
Overall	0.84	(0.76, 0.89)	0.92	(0.88, 0.94)
Prior to WTC exposure	0.91	(0.86, 0.94)	0.91	(0.86, 0.94)
During WTC exposure	0.59	(0.43, 0.71)	0.43	(0.25, 0.57)
After WTC exposure	0.75	(0.64, 0.82)	0.90	(0.86, 0.93)
Average Cigarette per Day				
Prior to WTC exposure	0.50	(0.33, 0.64)	0.72	(0.60, 0.81)
During WTC exposure	0.43	(0.25, 0.58)	0.71	(0.60, 0.80)
After WTC exposure	0.79	(0.69, 0.86)	0.65	(0.52, 0.75)
Ever Alcohol Drinking				
After WTC exposure	0.49	(0.27, 0.70)	0.53	(0.34, 0.71)

Note: WTC-CARES, World Trade Center Cancer Risk Epidemiology Study; WTCHP, World Trade Center Health Program; CI, confidence interval;

[a] Agreement assessed by Kappa (κ) statistic for categorical measures or intraclass correlation coefficient for continuous measures

Supplementary Table 3: WTCHP measures of behavioral risk factors among cases, by WTC-CARES enrollment status (N=102)

Construct	Enrolled, n=64 n (%)	Did Not Enroll, n=38 [a] n (%)	p-value [a]
Risk Behaviors			
Ever Cigarette Smoking			
Overall	33 (51.6)	18 (50.0)	0.8807
Prior to WTC exposure	33 (51.6)	18 (50.0)	0.8807
During WTC exposure	18 (28.6)	13 (36.1)	0.4365
After WTC exposure	15 (23.4)	12 (33.3)	0.2847
Years of Cigarette Smoking, mean ± SD			
Overall	11.1 ± 13.9	13.5 ± 16.2	0.4303
Prior to WTC exposure	10.1 ± 11.8	11.4 ± 14.2	0.6445
During WTC exposure	0.1 ± 0.2	0.2 ± 0.3	0.2530
After WTC exposure	1.5 ± 3.6	2.1 ± 4.3	0.4469
Average Cigarette per Day, mean ± SD			
Prior to WTC exposure	7.5 ± 9.6	7.2 ± 10.3	0.9005
During WTC exposure	4.4 ± 8.4	5.1 ± 9.0	0.7011
After WTC exposure	3.0 ± 7.2	4.9 ± 9.0	0.2385
Ever Alcohol Drinking			
After WTC exposure	38 (61.3)	22 (62.9)	0.8787

Note: WTCHP, World Trade Center Health Program; SD, standard deviation

[a] Includes 8 cases who were deceased prior to the start of the study

[b] 2-sided chi-square test for comparison of categorical measures, t-test for comparison of continuous measures
